# Low-intensity blue-enriched white light (750 lux) and standard bright light (10 000 lux) are equally effective in treating SAD. A randomized controlled study

**DOI:** 10.1186/1471-244X-11-17

**Published:** 2011-01-28

**Authors:** Ybe Meesters, Vera Dekker, Luc JM Schlangen, Elske H Bos, Martine J Ruiter

**Affiliations:** 1 University Center for Psychiatry, University Medical Center Groningen, Groningen, The Netherlands; 2 Philips Lighting, Eindhoven, The Netherlands; 3 Interdisciplinary Center for Psychiatric Epidemiology, University Medical Center Groningen, Groningen, The Netherlands; 4 University of Groningen, Department of Clinical and Developmental Psychology, Groningen, The Netherlands

## Abstract

**Background:**

Photoreceptor cells containing melanopsin play a role in the phase-shifting effects of short-wavelength light. In a previous study, we compared the standard light treatment (SLT) of SAD with treatment using short-wavelength blue-enriched white light (BLT). Both treatments used the same illuminance (10 000 lux) and were equally highly effective. It is still possible, however, that neither the newly-discovered photoreceptor cells, nor the biological clock play a major role in the therapeutic effects of light on SAD. Alternatively, these effects may at least be partly mediated by these receptor cells, which may have become saturated as a result of the high illuminances used in the therapy. This randomized controlled study compares the effects of low-intensity BLT to those of high-intensity SLT.

**Method:**

In a 22-day design, 22 patients suffering from a major depression with a seasonal pattern (SAD) were given light treatment (10 000 lux) for two weeks on workdays. Subjects were randomly assigned to either of the two conditions, with gender and age evenly distributed over the groups. Light treatment either consisted of 30 minutes SLT (5000°K) with the EnergyLight^® ^(Philips, Consumer Lifestyle) with a vertical illuminance of 10 000 lux at eye position or BLT (17 000°K) with a vertical illuminance of 750 lux using a prototype of the EnergyLight^® ^which emitted a higher proportion of short-wavelengths. All participants completed questionnaires concerning mood, activation and sleep quality on a daily basis. Mood and energy levels were also assessed on a weekly basis by means of the SIGH-SAD and other assessment tools.

**Results:**

On day 22, SIGH-SAD ratings were significantly lower than on day 1 (SLT 65.2% and BLT 76.4%). On the basis of all assessments no statistically significant differences were found between the two conditions.

**Conclusion:**

With sample size being small, conclusions can only be preliminary. Both treatment conditions were found to be highly effective. The therapeutic effects of low-intensity blue-enriched light were comparable to those of the standard light treatment. Saturation effects may play a role, even with a light intensity of 750 lux. The therapeutic effects of blue-enriched white light in the treatment of SAD at illuminances as low as 750 lux help bring light treatment for SAD within reach of standard workplace and educational lighting systems.

## Background

Exposure to bright light has proved to be a very effective treatment for seasonal affective disorder (SAD), winter type, for over 25 years now [1-3]. Ever since light treatment was first used, light fixtures and treatment models have improved and have followed science-based innovations. A recent scientific development has been the discovery of a novel photoreceptor, melanopsin, within the basal ganglia of the retina [4-6]. This new photoreceptor plays a major role in regulating the biological clock [5,7] and is also involved in pupillary constriction [8]. It influences the circadian system and is the most sensitive to light with a wavelength of about 480 nm (blue light) [9-11]. According to the phase-shift hypothesis, the biological clock is very important in the aetiology of SAD and the working mechanisms of light treatment [12]. According to this hypothesis, blue light is thought to be more powerful in the treatment of SAD than light of other wavelengths. In the treatment of SAD, exposure to blue narrow-band light with an intensity of 398 lux was in fact, found to be superior to dim red-light therapy of 23 lux [13]. Equally, narrow-band blue light of a lower intensity (176 lux) was found to be superior to narrow-band red light of 201 lux [14].

In a previous study, however, we failed to find any differences in treatment outcome after exposure to standard light treatment (SLT) and blue-enriched light treatment (BLT) of identical intensities. A possible explanation of this result may be that the maximum (saturated) response to light treatment occurs at the illuminance (~10000 lux) used in the comparison. Adding more short-wavelength light can not increase this response any further [15]. When equating the short wavelength (424-532 nm) photon density at lower illuminances, blue monochromatic light of a modest photopic intensity (98 lux) was equally effective in treating SAD as white light of 711 lux [16].

This suggests that low-intensity light treatment, either by blue light alone, or by blue-enriched white light may be just as effective as the high-intensity lights used in the SLT devices. In this study, we compared the effects of the treatment of SAD after exposure to low-intensity BLT (750 lux) to those after exposure to high-intensity SLT (10000 lux).

Apart from a depressed mood, lack of energy and decreased levels of activity and sleep quality are well-known symptoms in patients suffering from SAD [1]. We therefore assessed the effects on mood, energy, different aspects of activation, and sleep quality in two conditions.

## Methods

### Subjects

In the winter of 2008-2009 patients of the SAD outpatient clinic of the University Medical Center Groningen, the Netherlands were asked to participate in the study. Eight patients were recruited by means of an advertisement in a local newspaper. Potential participants were sent written information and were invited for an intake interview at the clinic in order to obtain a diagnosis by an experienced clinical psychologist. The participants were informed about the goal of the study: to investigate the effects of low-intensity blue-enriched light treatment compared to standard light treatment. If patients were suffering from winter depression, they were given information about the research project. After they had signed the informed consent form, a screening visit was scheduled. Twenty-three patients were included in the study. After a few days of light treatment, one patient dropped out for reasons unrelated to her depression (concussion) and was excluded from the study, leaving 22 patients. In the SLT condition 3 men and 8 women participated (mean age 39.9 yrs ± 12.7), in the BLT condition this amounted to 2 men and 9 women (mean age 41.7 yrs ± 13.1).

The research protocol was approved by the Medical Ethical Committee of the University Medical Center Groningen.

### Light therapy

Light treatment consisted of 2 weeks of SLT (correlated colour temperature 5000°K, vertical illuminance at eye position: 10 000 lux) with the EnergyLight (Philips Consumer Lifestyle B.V., Drachten, The Netherlands) or 2 weeks of BLT (correlated colour temperature 17 000°K) with a vertical illuminance of 750 lux at eye position, administered with an EnergyLight equipped with a special prototype lamp emitting white light with a high proportion of short wavelengths with a correlated colour temperature of 17 000°K, as in the Philips ActiViva Active lamps. An international standard for retinal blue-light hazard risk has been defined to protect participants against retinal photochemical injury from chronic blue-light exposure [17]. All light conditions used in this study remain far below the exposure limits as defined by this standard.

**Figure 1:**
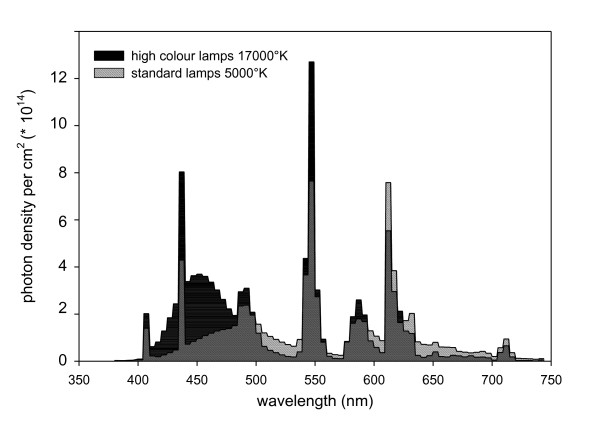
**Spectral-power distributions of the lamps used in the standard EnergyLight^® ^(5000°K) and the specially-prepared EnergyLight^® ^with blue-enriched white light (17 000°K)**.

During light therapy, patients were sitting at equal distances (20 cm) from the EnergyLight in both conditions. In Figure 1 the spectral-power distributions of the standard light and of the blue-enriched white light lamps are shown.

Subjects came to the university hospital on 10 workdays (days 4-8 and days 11-15 in the protocol) and were given either 30 minutes SLT or BLT between 7:45 and 8:45 a.m. Treatment was given to one patient at a time, without staff or other people being present. The SLT condition had an illuminance of 10 000 lux and a correlated colour temperature of 5000 °K. In the 380-740 nm wavelength band the SLT irradiance was 3207 microW/cm2, with a total photon flux of 8.84 × 10^15 ^photons/cm^2^/s. The SLT irradiance within the 424-532 nm band equals 1135 microW/cm2, with a photon flux of 2.7 × 10^15 ^photons/cm^2^/s. The BLT illuminance equals 750 Lux, with a correlated colour temperature of 17 000 °K. The BLT irradiance within the 380-740 nm range equals 307 microW/cm2, with a total photon flux of 7.81 × 10^14 ^photons/cm^2^/s. The BLT irradiance within the 424-532 nm band equals 168 microW/cm2, with a photon flux of 3.9 × 10^14 ^photons/cm^2^/s.

### Assessment and procedure

During the screening visit, patients were assessed by means of a standardized structured interview, the Mini-International Neuropsychiatric Interview (M.I.N.I) [18]. Subjects meeting the criteria of a major depressive disorder, seasonal pattern, winter type, according to the DSM-IV-TR [19] were subsequently assessed by means of the Structured Interview Guide for the Hamilton Depression Rating Scale-Seasonal Affective Disorder, 24-item version (SIGH-SAD) [20]. After that they were asked to complete the Beck Depression Inventory-II-NL (BDI-II-NL) [21], and a questionnaire aiming to evaluate subjects' expectations of the effects of light therapy. On a 5-point scale this questionnaire rated both for SLT and BLT whether subjects expected to benefit from the therapy, whether they thought it was a logical treatment, and whether they would recommend this therapy to a friend. Subjects who met all inclusion criteria were randomly assigned to one of the two conditions, with gender and age distributed evenly over the groups. They were not told which of the two conditions they were going to participate in.

Each of the two conditions started at day 1 (Friday) with a baseline measurement consisting of a SIGH-SAD interview, the BDI-II-NL and a fatigue self-rating questionnaire (Short Fatigue Questionnaire, SFQ) [22]. The SIGH-SAD interviewers were unaware of the experimental conditions. The SIGH-SAD, the BDI-II-NL and SFQ were repeated at day 8 (directly after the 5^th ^light session), at day 15 (directly after the 10^th ^light session) and at day 22 (1 week after light treatment had ended).

On day 22, an evaluation questionnaire was added to check the outcome of subjects' expectations on day 1. Furthermore, subjects were asked which condition they thought they had been treated in.

Starting at day 1, before 8.00 a.m. and at least 30 minutes after waking up subjects rated their mood and sleep quality of the previous night on a daily basis using the Adjective Mood Scale (AMS)[23,24] and the Groninger Sleep Quality Scale (GSQS)[25,26]. Also, the following four components of activation were measured, using the Activation-Deactivation Check List (AD-ACL)[27]: General Activation (GA; i.e. energetic, vigorous, full of pep, active, and lively), Deactivation-Sleep (DS; i.e. sleepy, tired, drowsy, wide awake, and wakeful), High Activation (HA; i.e. jittery, intense, fearful, clutched-up, and tense), and General Deactivation (GD; i.e. placid, calm, at rest, still and quiet). To describe their current feelings, subjects were asked to rate these 20 adjectives on a 4-point scale. The scores of the first 3 days on the daily questionnaires (before light treatment) were considered baseline.

### Statistics

Baseline differences between the two conditions were tested by means of t-tests (continuous outcomes) and chi-square tests (dichotomous outcomes).

Effect sizes [28] were calculated for each condition. These effect sizes reflect the differences between baseline (day 1) and day 22. Results were based on the weekly assessments of the two conditions and were compared by means of repeated measures ANOVA. This was done for the patients who had complete data for these measures (n= 11 vs.11).

Linear Mixed Models were used to compare the two conditions on the basis of the daily self-rating questionnaires.

An advantage of these models is that all available data can be used, including those of subjects with one or more missing values. Consequently, in these analyses data of all 22 subjects were used. Another advantage of linear mixed models is that they allow for including random effects; i.e. parameters are allowed to vary across individuals. This may reveal heterogeneity in individual growth curves. We used models with time, condition, and the interaction between time and condition, with the baseline score as a covariate (baseline score = mean of the 3 pre-intervention scores). We fitted models with the 22 days as the repeated measures and allowed the slope to vary across individuals. Maximum likelihood estimation was used. We compared models with different variance-covariance matrices. Selection of the final model was based on the Bayesian Information Criterion (BIC; with lower values indicating better models). If the random effect for slope was found to be non-significant, this term was removed from the model (unless this resulted in a higher value of the BIC criterion). Regression assumptions were checked by performing residual diagnostics on the final models.

In a secondary analysis, we examined the potential impact of the initial severity of the complaints on outcome. To this end, we added the interaction baseline*time to the models. We also examined whether this effect of baseline severity differed for the different conditions by adding the interaction condition*baseline*time to the models, including all lower-order terms.

A responder was defined as a subject who improved at least by 50%. Analyses were carried out using SPSS 17. A two-tailed alpha level of 0.05 was used to determine statistical significance.

## Results

At the start of the experiment, there was no statistical difference between the conditions with regard to gender, age, and severity of depression or other complaints and expectations about the effects of the light conditions as measured by the self-rating questionnaires or standardized interviews. All 22 participating subjects received the intervention as intended.

### Weekly assessments

The results of the weekly assessment procedures are summarized in Table 1. Although subjects in both conditions improved after exposure to light treatment, there were no statistical differences between these improvements.

**Table 1:** Weekly average depression scores (±SD)

Condition		N	Day 1 (SD)	Day 8 (SD)	Day 15 (SD)	Day 22 (SD)	Effect size d	% Response	Responder N
SIGH-SAD	SLT	11	25.6 (6.3)	18.1 (8.0)	10.9 (4.4)	8.9 (6.8)	2.54	65.2	8
	BLT	11	25.4 (6.9)	19 (6.0)	14 (10.1)	6 (4.0)	3.53	76.4	11
HRSD	SLT	11	13.8 (4.6)	9.9 (4.3)	6.8 (3.0)	4.9 (3.9)	2.09	64.5	9
	BLT	11	13,7 (5.4)	10,45(3.3)	7.8 (4,9)	3.4 (2.0)	2.53	75.2	10
ATYP	SLT	11	11.8 (4.6)	8.2 (4.3)	4 (2.2)	4 (3.5)	1.91	66.1	8
	BLT	11	11.6 (3.5)	8.5 (4.3)	6.2 (5.8)	2.6 (2.5)	2.96	77.6	10
BDI-II	SLT	11	23 (5.3)	15.3 (8.1)	9.1 (6.4)	7.1 (6.4)	2.71	69.1	9
	BLT	11	26.6 (10.8)	17.8 (11.7)	14.9 (11.4)	5.3 (3.8)	2.63	80.1	10
SFQ	SLT	11	24.6 (2.0)	20.5 (7.0)	16.5 (6.0)	14.2 (5.5)	2.51	42.3	4
	BLT	11	25.2 (2.8)	20.2 (6.2)	18.5 (7.5)	11 (4.5)	3.79	56.3	8

Cohen's d effect size and response percentage from day 1 to day 22, as rated by the Hamilton Rating Scale for Depression (HRSD, 17 items), the scale that has been adapted for seasonal symptoms SIGH-SAD (24 items), and the atypical symptoms separately ATYP (7 items), the score on the Beck Depression Inventory-II and the Short Fatigue Questionnaire for each condition. SLT = standard light treatment; BLT= blue-enriched white-light treatment

Responder = subject with an improvement of at least 50%

In both conditions, depressive complaints decreased during the 3-week period (Table 1, SIGH-SAD 24 items, main effect "time" F(3,18) = 30.2, p < 0.001), with no significant differences between conditions (main effect "condition" F(1,20) = 0.012, ns) nor over time between conditions (interaction effect "time*condition" F(3,60) = 0.95, ns). The same pattern emerged when the SIGH-SAD was subdivided into "typical symptoms" (17-item Hamilton rating, Table 1, main effect "time" F(3,18) = 28.2, p < 0.001; main effect "condition" F(1,20) = 0.00, ns; interaction effect "time*condition" F(3,60) = 0.62, ns) and "atypical items" (7 atypical items, Table 1, main effect "time" F(3,18) = 18.84, p < 0.001; main effect "condition" F(3,20) = 0.039, ns; interaction effect "time*condition" F(3,60) = 0.99, ns). Calculations based on the BDI-II-NL scores showed similar results (main effect "time" F(3,18) = 31.4, p < 0.001; main effect "condition" F(1,20) = 0.78, ns; interaction effect "time*condition" F(3,60) = 1.57, ns). Calculations based on the SFQ scores point in the same direction (main effect "time" F(3,18) = 39.6, p < 0.001; main effect "condition" F(1,20) = 0.21, ns; interaction effect "time*condition" F(3,60) = 1.42, ns). Although the number of responders differs in the two conditions (measurements based on the weekly ratings), this difference was not statistically significant.

The evaluation questionnaire taken at day 22 shows that participants of the blue-enriched white-light condition experienced the treatment as less comfortable than participants of the standard bright-light treatment (F(1,20) = 9.61, p = 0.006). After treatment, 2 subjects in the SLT condition thought they had been treated in the other condition, another 2 were unsure. In the BLT condition 1 subject thought he had been treated in the other condition and another one was unsure.

### Daily questionnaires

**Figure 2:**
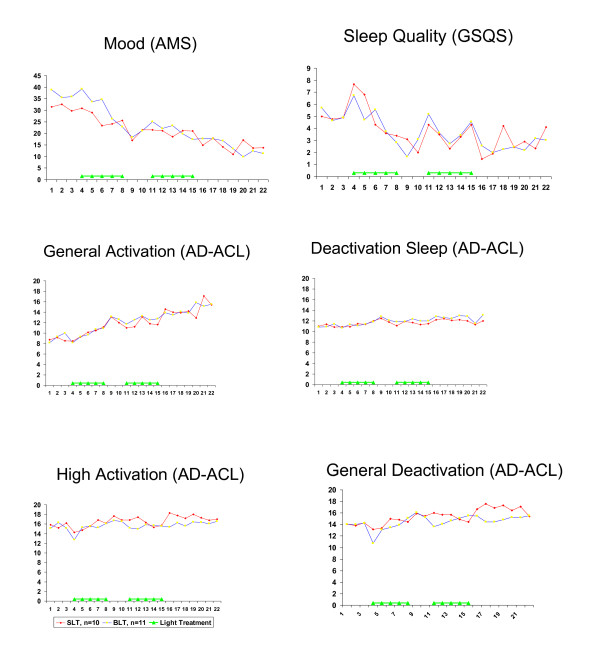
**Scores on daily self-rating questionnaires assessing mood, sleep quality and four aspects of activation**. For abbreviations: see text. Higher scores on the AMS and GSQS mean more symptoms; higher scores on the AD-ACL mean fewer complaints.

As can be seen from Table 2, the results of the daily self-rating questionnaires are in line with the results of the weekly assessment procedures. There was no statistically significant difference in the way subjects improved. The interaction time*condition was not significant in any of the models. The time effect, on the other hand, was significant in all models. Thus, mood, sleep quality and energy levels improved in both conditions (Figure 2 and Table 2).

**Table 2:** Daily self-rating questionnaires.

Outcome	Model	Estimate	P-value
Mood (AMS)	Time	-0.946	.000
	condition	3.593	.283
	time*condition	-0.361	.152
	baseline	0.619	.000

Sleep (GSQS)	Time	-0.154	.000
	condition	-0.054	.912
	time*condition	0.008	.880
	baseline	0.570	.000

Deactivation Sleep (AD-ACL)	Time	0.058	.031
	condition	-0.045	.857
	time*condition	0.028	.440
	baseline	0.601	.000

General Activation (AD-ACL)	Time	0.257	.000
	condition	-0.124	.894
	time*condition	0.036	.606
	baseline	0.472	.000

High Activation (AD-ACL)	Time	0.090	.039
	condition	-0.220	.583
	time*condition	-0.020	.730
	baseline	0.887	.000

General Deactivation (AD-ACL)	Time	0.148	.001
	condition	-0.282	.471
	time*condition	-0.045	.440
	baseline	0.813	.000

Results of regression analyses

We also examined the influence of gender and age on the outcomes. No interaction effects were found between gender or age on the one hand and time or time*condition on the other. Adjustments for gender and age did not cause any substantial changes in the results either. Therefore, gender and age have not been included in the final models.

### Impact of baseline severity on outcome

As can be seen from Table 2, in all models baseline severity was related to outcome, with higher baseline scores predicting higher overall follow-up scores. The interaction between baseline scores and time was significant in the models for Mood (p < 0.001), Sleep (p < 0.05), General Activation (p < 0.005), and General Deactivation (p < 0.05). Consequently, baseline severity was also related to change in symptoms over time. The regression coefficients for the interaction effects had a negative sign, which implies that higher baseline severity predicted steeper slopes (more improvement).

The interaction condition*baseline*time was non-significant in all models. This indicates that the effects of baseline severity on outcome did not differ for the different conditions.

## Discussion

The sample size in this study is very small, so results, though promising, can only be very preliminary.

On all parameters, the effects of exposure to low-intensity blue-enriched white light in the treatment of SAD did not differ from the effects of exposure to standard bright-light treatment. In this study, participants experienced blue-enriched white light as less comfortable than the standard light condition. In a previous SAD treatment study using blue-enriched white light of higher intensities, no differences in appreciation emerged between BLT and SLT [15].

However, in this study, participants indicated they found the lower intensity blue-enriched white light slightly less pleasant than the standard light condition, a finding based on their answers on the evaluation questionnaire. In the SLT group 63% (7 out of 11) of the subjects rated the treatment as "rather pleasant" or "very pleasant". In the BLT group, treatment appreciation was slightly lower: 81% (9 out of 11) of the subjects in the BLT group indicated the treatment to be "neither pleasant, nor unpleasant", "rather pleasant" or "pleasant".

This small difference in appreciation in the present study is significant but must be interpreted within the context of the relatively modest sample size.

Moreover, in office settings, illuminances of around 360 lux, blue-enriched white light (17 000 °K) have been reported to improve subjective measures of irritability and eye discomfort as compared to white light of 4000 °K [29].

Exposure to low-intensity blue-enriched white light (750 lux, 17 000 °K) is equally effective as standard full-spectrum light treatment (10 000 lux, 5000 °K). It would also have been interesting to make a direct comparison between the effects of exposure to standard light at 750 lux and at 10 000 lux. However, data of other studies indicate that higher-intensity light treatment leads to larger improvements than light treatment with lower-intensity light [2,30].

Results are in line with those of Anderson et al. [16], who found that blue monochromatic low-intensity light (98 lux) was equally effective in treating SAD as broadband white light at 711 lux with identical photon density in the 424-532 nm range. The short wavelength photon flux of these two conditions is highly comparable to the BLT condition of the present study. The current findings on exposure to low-intensity blue-enriched white light are also in line with the results of studies in office surroundings. It has been shown that sleep quality and alertness improved when workers spent their day in an office with blue-enriched white light with an intensity of 310.35 lux in the daytime instead of in the standard white (4000 °K) office lighting conditions with a mean intensity of 421.07 lux [29]. In a similar study, the standard room lighting had an intensity of 345 lux and was compared to blue-enriched light with an intensity of 354 lux [31].

As the biological clock is known to be highly sensitive to blue light, the phase-shift hypothesis suggests that blue-enriched light is a more powerful treatment for SAD than standard light. Although exposure to low-intensity blue-enriched white light in SAD patients leads to the same therapeutic results as exposure to the standard bright-light treatment, this does not necessarily indicates that blue or blue-enriched light has a more powerful influence on the biological clock. Studies by Smith et al. [32,33], have demonstrated that, in a similar way, frequently used bright-light therapy photon densities (4.2 vs. 4.9 10E15 photons/cm2/s), blue-enriched white light (17 000 °K) does not outperform standard white light (4100 °K) in phase-advancing or phase-delaying effects. Interestingly, a recent study indicated that for irradiances between 2E12 and 1.5E14 photons/cm2/s, blue (460 nm) light does in fact outperform green light (555 nm) in phase-shifting effects, whereas blue light yields smaller phase-shifts than green light of identical photon density at lower intensities (in the 2.5E11-2E12 photons/cm2/s range) [34].

An alternative explanation for our finding that the effects of blue-enriched light are not better than those of standard light is the possibility that the blue wavelengths are not necessary for the therapeutic effects of the treatment of SAD. Blue light plays a role in the working mechanism of the biological clock, but the role of the biological clock itself in the aetiology of SAD has not been fully established yet [35-37].

Since this study has no placebo condition included, the similar responses to the two treatments could be interpreted as placebo effects only. It is impossible to create a real placebo condition for visible light treatment, though. The few studies testing light therapy in winter depressives using some kind of placebo condition (for example a deactivated negative-ion generator) revealed placebo effects that ranged from 21% to 41% [38-40]. In a placebo-controlled study of extra-ocular light treatment, we found a placebo response of 36% [41]. In this latter study, participants visited the clinic in the mornings for treatment, which was similar to the visits in this study. The response rates in the current study between 65% and 76% for remission are relatively high compared to the placebo responses from the placebo-controlled studies, probably too high to be interpreted as placebo effects only, although we can not rule out this possibility.

## Conclusion

Although the role of blue light in the treatment of SAD is still unclear, low-intensity blue-enriched white light with an intensity of 750 lux is highly effective, and equal to standard bright light at 10 000 lux, 5000 °K. Monochromatic light of even lower intensity has also shown to be effective in treating SAD [16]. At present it is unknown at what light intensities the SAD light-therapy response reaches saturation: this level may be lower than the blue-enriched white-light setting of 750 lux which is currently being used. Therefore, it is possible that blue-enriched white light with intensities below 750 lux still yields the same beneficial effects. Further work is needed to investigate whether an intensity threshold can be established for light treatment for SAD and to find the lowest possible light intensity that makes optimal treatment possible. As indicated in the current findings, this lowest effective intensity may depend on the spectral characteristics of the light source. Further research is needed to find the lowest optimal intensity for blue-enriched white light. If blue-enriched white light with intensity below 750 lux is found to be effective in treating SAD, this may make it possible to use this light in the regular room lighting fixtures of patients, or even in general lighting systems used in workplaces and in educational and healthcare settings.

## Competing interests

YM has received research funding and served as a consultant for Royal Philips Electronics NV and The Litebook Company Ltd.; LJMS is an employee of Philips Lighting. VD; EHB and MJR reported no potential conflicts of interest.

## Authors' contributions

The original version of the experimental protocol was written by YM, LJMS and MJR. YM served as principal investigator. VD participated in the clinical conduct of the trial and was the research coordinator. EHB contributed to the statistical data analysis. The final manuscript was written by YM, with comments of all co-authors, all of whom read and approved the final manuscript.

## Pre-publication history

The pre-publication history for this paper can be accessed here:

http://www.biomedcentral.com/1471-244X/11/17/prepub
